# Hidden populations: risk behaviours in drug-using populations in the Republic of Georgia through subsequent peer-driven interventions

**DOI:** 10.1186/s12954-021-00527-y

**Published:** 2021-07-28

**Authors:** Cale Lawlor, Marine Gogia, Irma Kirtadze, Keti Stvilia, Guranda Jikia, Tamar Zurashvili

**Affiliations:** 1 The Georgian Harm Reduction Network, Tbilisi, Georgia; 2 Alternative Georgia, Tbilisi, Georgia; 3 National Centre for Disease Control and Prevention, Tbilisi, Georgia

**Keywords:** Drug use, Drug risk, Sex risk, Harm reduction, HIV, Hepatitis, Needle exchange, Peers, Peer recruitment, Peer network

## Abstract

**Background:**

Georgia has a significant risk of ongoing HIV and HCV outbreak. Within this context, harm reduction aims to reduce risk associated with drug use through community activities, such as peer recruitment and involvement. The aim of this study was to identify significant differences between known and hidden populations, and attest to the ongoing utility of peer-driven intervention across multiple years in recruiting high-risk, vulnerable populations through peer networks. It was hypothesised that significant differences would remain between known, and previously unknown, members of the drug-using community, and that peer-driven intervention would recruit individuals with high-risk, vulnerable individuals with significant differences to the known population.

**Methods:**

Sampling occurred across 9 months in 11 cities in Georgia, recruiting a total of 2807 drug-using individuals. Standardised questionnaires were completed for all consenting and eligible participants, noting degree of involvement in harm reduction activities. These data underwent analysis to identify statistically significant different between those known and unknown to harm reduction activities, including in demographics, knowledge and risk behaviours.

**Results:**

Peer recruitment was able to attract a significantly different cohort compared to those already known to harm reduction services. Peer-driven intervention was able to recruit a younger population by design, with 25.1% of PDI participants being under 25, compared to 3.2% of NSP participants. PDI successfully recruited women by design, with 6.9% of PDI participants being women compared to 2.0% in the NSP sample. Important differences in drug use, behaviour and risk were seen between the two groups, with the peer-recruited cohort undertaking higher-risk injecting behaviours. A mixture of risk differences was seen across different subgroups and between the known and unknown population. Overall risk, driven by sex risk, was consistently higher in younger people (0.59 vs 0.57, *p* = 0.00). Recent overdose was associated with higher risk in all risk categories. Regression showed age and location as important variables in overall risk. Peer-recruited individuals reported much lower rates of previous HIV testing (34.2% vs 99.5%, *p* = 0.00). HIV knowledge and status were not significantly different.

**Conclusions:**

Significant differences were seen between the known and unknown drug-using populations, and between previous and current research, speaking to the dynamic change of the drug-using culture. The recruitment strategy was successful in recruiting females and younger people. This is especially important, given that this sampling followed subsequent rounds of peer-driven intervention, implying the ability of peer-assisted recruitment to consistently reach hidden, unknown populations of the drug-using community, who have different risks and behaviours. Risk differences were seen compared to previous samples, lending strength to the peer-recruitment model, but also informing how harm reduction programmes should cater services, such as education, to different cohorts.

## Introduction

People who inject drugs (PWIDs) have an increased risk of HIV and hepatitis C (HCV) infection, along with the morbidity and mortality associated with this. While there are many effective interventions that can be used in this population, in the field of harm reduction, needle and syringe programmes (NSPs) can help to lower the risk associated with injecting drug use. While NSPs can be effective in recruiting PWIDs to their services, others remain outside of the reach of NSP services, hidden to public health efforts. Peer-driven interventions (PDIs), including peer recruitment and respondent-driven sampling (RDS), have been shown to be effective way to recruit those outside of the reach of services, leading to engagement with harm reduction services, and the benefits that come with engagement [1].

The Republic of Georgia is at considerable public health risk from HIV and HCV due to injecting drug use. The last figures available, from 2016, estimate that 1.41% of the Georgian population partake in injecting drug use, with a 2.24% prevalence in the 15–64 age group [2]. These figures applied to the Georgian population estimates at the start of 2020 would estimate a number of just over 52700 [3]. There is no update to date survey on the size of this population. The most recent number of officially registered cases of HIV/AIDS stood at 8299 (0.22% of the population), though it is estimated that the true figure is higher [4]. Of these, 37.3% are believed to have been attributed to injecting drug use [4]. The statistics are higher for HCV infection; a prevalence study from 2016 estimated HCV antibodies to be present in 7.7% of the Georgian population, with 5.4% testing positive for active infection [5]. PWIDs remain a particularly high-risk group for HCV infection, with one-third of the figures in the general population thought to be related to injecting drug use [6] and with up to 75% of PWIDs exposed to HCV [7]. Like with HIV, Georgia is particularly vulnerable to HCV epidemic [8]. Drug use is criminalised in Georgia [9].

NSPs have been active in Georgia since 2005. A number of local non-government organisations (NGOs) conduct local harm reduction activities, including the Georgian Harm Reduction Network (GHRN), the authors of this report. In 2019, GHRN provided just under 4 million syringes across its reach of 35,800 clients, which equalled 76 syringes per client per year, far below the WHO recommendation of 300 per client per year [10]. In 2019, just over 28,000 clients were tested for HIV, as well as 2400 family members of clients. These programmes have proved successful in attracting people in the drug-using community to harm reduction services, evidenced by growing engagement with services.

PDIs recruit clients known to harm reduction programmes and encourage those clients to recruit people in their social or community network to harm reduction activities, and are utilised in Georgia. GHRN published a study report around a PDI in 2019, based on respondent-driven sampling among PWIDs conducted in 2015 [11]. The PDI analysed was able to recruit previously hidden drug-using populations, with statistically different demographics, risk behaviours and knowledge to the NSP sample. Statistically significant differences were found, with the peer respondent group having higher rates of unemployment, lower rates of home ownership, younger age and a higher proportion of people identifying as homosexual [11]. They were more likely to exchange sex for drugs or money and less likely to “always” use a condom [11]. Those recruited by respondent-driven sampling started injecting at a younger age and shared syringes and equipment more frequently [11]. They were dramatically less likely to have ever been tested for HIV, were less likely to have been recently tested for HIV, less likely to know their HIV status and scored lower on measures of HIV knowledge [11]. The PDI was very successful in recruiting diverse populations of the drug-using community to harm reduction activities that represent particularly high risk.

Regional and international literature concurs; PDIs can recruit younger PWIDs, with significant differences in knowledge and risk-taking, and can reduce injection frequency, reduce rates of syringe and equipment sharing, and reduce rates of unprotected sex, overall, reducing health risk [1]. PDI is an extremely useful tool in helping control blood-borne disease and drug use-associated health risk [1]. PDIs are able to reach a more diverse drug-using population [12].

Literature published since the research of the previous report has gone on to lend more strength to the importance of PDIs. The use of peers to engage the IDU population was seen to increase trust of the staff running harm reduction activities among the recruited, which can counteract the barrier of criminalisation, if it is present [13]. Trust was identified as an important facilitator, especially when aiming to recruit vulnerable minority groups, and this trust was associated with a decrease in stigma and increase in ongoing engagement with harm reduction programmes [14]. Financial incentives were a motivator, but not the only motivator; knowledge gaining was also seen as a benefit of those newly recruited [13]. The use of peers in programmes focussing on PWIDs and HCV found that they can act as a “bridge” between harm reduction programmes and peer networks and can play a central role in harm reduction efforts [15]. They can also help to identify people previously engaged in harm reduction programmes who have since dropped out [16]. Peers have a surprising amount of social connection within their population, with a modelling showing that engaging just 5.6% of the drug-using community could reach 70% of a drug-using population through peer recruitment [17]. The modelling also demonstrated that peer-guided recruitment was able to reach vulnerable users who sat on the “periphery” of social networks [17]. Peer-driven interventions have also been further quantified to be cost-effective [18].

Since the pilot programme, PDIs have gone on to be used as a recruitment method for PWIDs in Georgia. This report details of sampling of peer-recruited PWIDs against previously known NSP clients, to identify the ongoing significance of differences between populations as PDI recruits further into drug-using populations. The purpose is to show that multiple rounds of PDIs with respondent-driven sampling are useful in continually recruiting previously hidden populations, further to previous successes, as well as prove the efficacy of PDIs with RDS to recruit vulnerable, high-risk populations. To our knowledge, no research has analysed differences in populations, and their vulnerabilities and risk, through multiple rounds of PDI. We hypothesise that important, significant differences will continue to exist between the two populations.

## Methods

Two comparative cross-sectional surveys with convenience sampling in the NSP group and respondent-driven sampling in the PDI group were conducted. NSP clients were already recruited to, and familiar with, harm reduction activities. The PDI sample was recruited by peers. Special incentive was given to recruit women and young people (under 25) by RDS methods, to recruit more vulnerable populations to harm reduction activities. An ethics framework was produced and submitted to ensure that all participants were voluntary and competent to make an informed decision and that confidentiality was protected as much as practicable. Sampling was performed over a 9-month period between October 2018 and June 2019 in 11 cities in Georgia (Tbilisi, Batumi, Akhaltsikhe, Rustavi, Kutaisi, Zugdidi, Telavi, Ozurgeti, Samtredia, Poti and Borjomi). The use of a multi-centre approach was both to gain a robust sample size, but also to guarantee diversity of the samples through reducing bias through affiliation (for example, homophily or heterophily). A trained consultant of voluntary counselling and testing (VCT) carried out the standardised questionnaire. RDS sampling through peer networks continued until sample size was adequate, and not exceeding 2600 participants. Sample size was constrained by budget, consent to participate and time. All those eligible for participation in the survey were included. The total number sampled in both groups was a product of these factors, with the aim to recruit as many as possible. Eligibility criteria included:

1. Older than 18 years old
2. Participation on voluntary grounds
3. Competent to consent and participate in study (in reference to medical conditions and mental health conditions)
4. Minimum 6-month involvement with NSP program (NSP participants only)/non-participation in HIV prevention programmes, including NSP, in the year prior (PDI participants only)
5. Recruited by peer as part of PDI activity, with coupon (PDI participants only)
6. Presence of drug injection track marks (PDI participants only, as high-likelihood evidence of current or recent injecting drug use)

The PDI started with NSP “seed” participants who were incentivised to educate and recruit peers previously unknown to the programme. As used typically in RDS, coupons were issued to the “seeds” for distribution and recruitment through their peer network. A minimum of 2 and maximum of 9 recruiters were used as “seeds” in each city, a total of 54. On first introduction, new PWIDs were offered to participate in sampling and to become involved as recruiters. They were offered an education session, and then, any PWIDs they recruited (linked by a coupon number) were tested on knowledge. Education session included HIV transmission, window periods, injected drug-related harm, homemade drugs, overdose and first aid, tuberculosis, sexually transmitted infections and viral hepatitis. Peer education could occur through whatever method the recruiter chose, and this would usually be through social interaction with partners and friends, during outreach, or during drug cooking. No new PWIDs who were not recruited by a peer were included in the PDI sample. Financial incentives were provided in reference to knowledge of topics covered in original education session with new recruits, with 1 Lari (GEL, €0.25) provided for each correct answer, as are often used in RDS. Incentives were also provided for time and transport costs. If the second-generation recruit did not score any correct answers in knowledge, their recruiter was not provided further recruitment coupons (though they were also offered follow-up services and education). If the respondent answered questions correctly, the first-generation recruiter was offered 3 further coupons. Incentives were provided based on the number of correct answers, up to 30 GEL (€7.6). Extra incentives were given for recruitment of the target subgroups of women and young people who inject drugs (5 GEL, €1.3). Ten GEL (€2.6) was provided for completing the survey questionnaire. All PWIDs were offered harm reduction services.

**Fig. 1:**
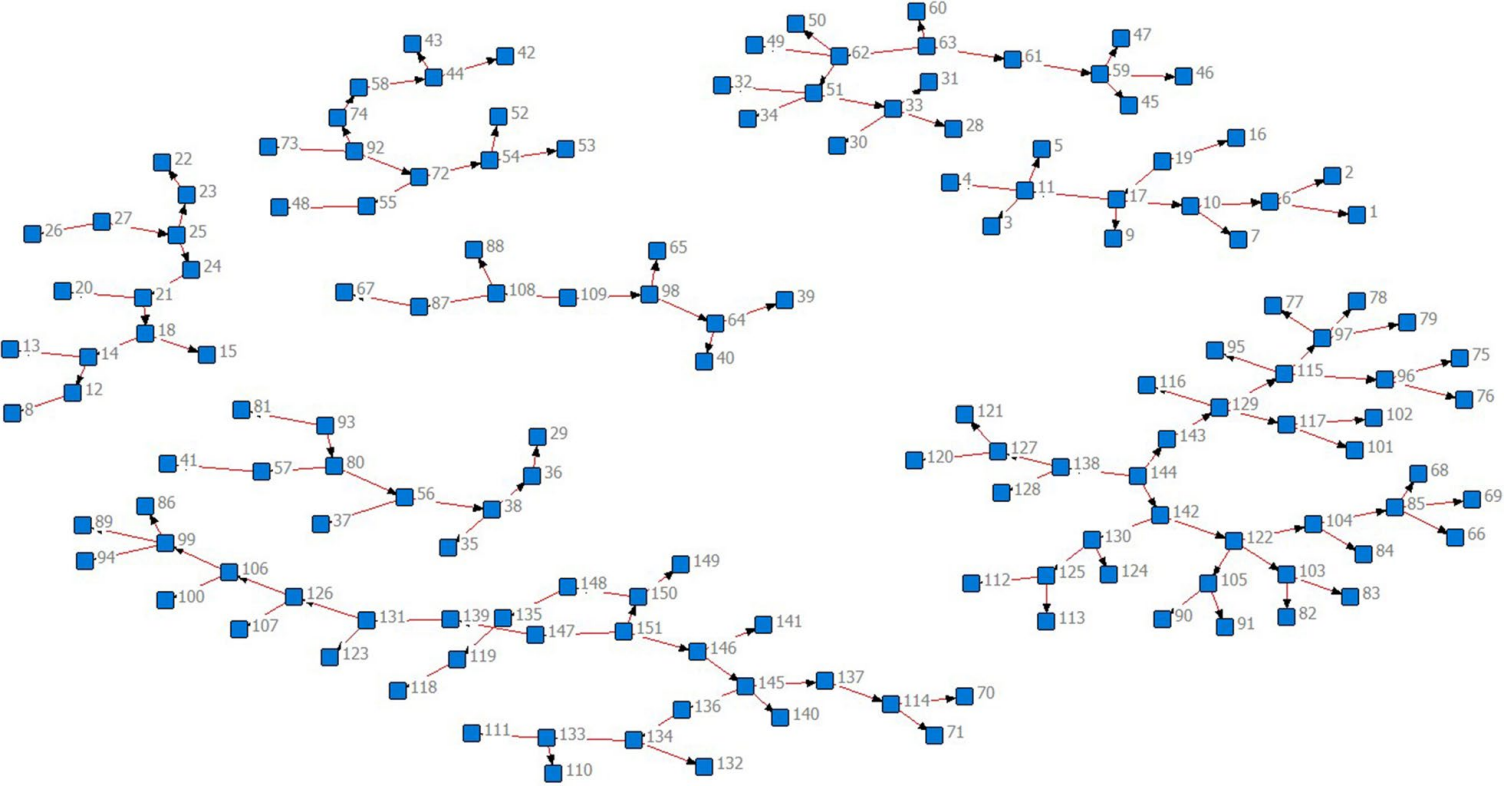
Peer-driven recruitment network from initial seed clients as recruited by the harm reduction organisation “New Vector”, a VCT NGO based throughout Georgia

**Fig. 2:**
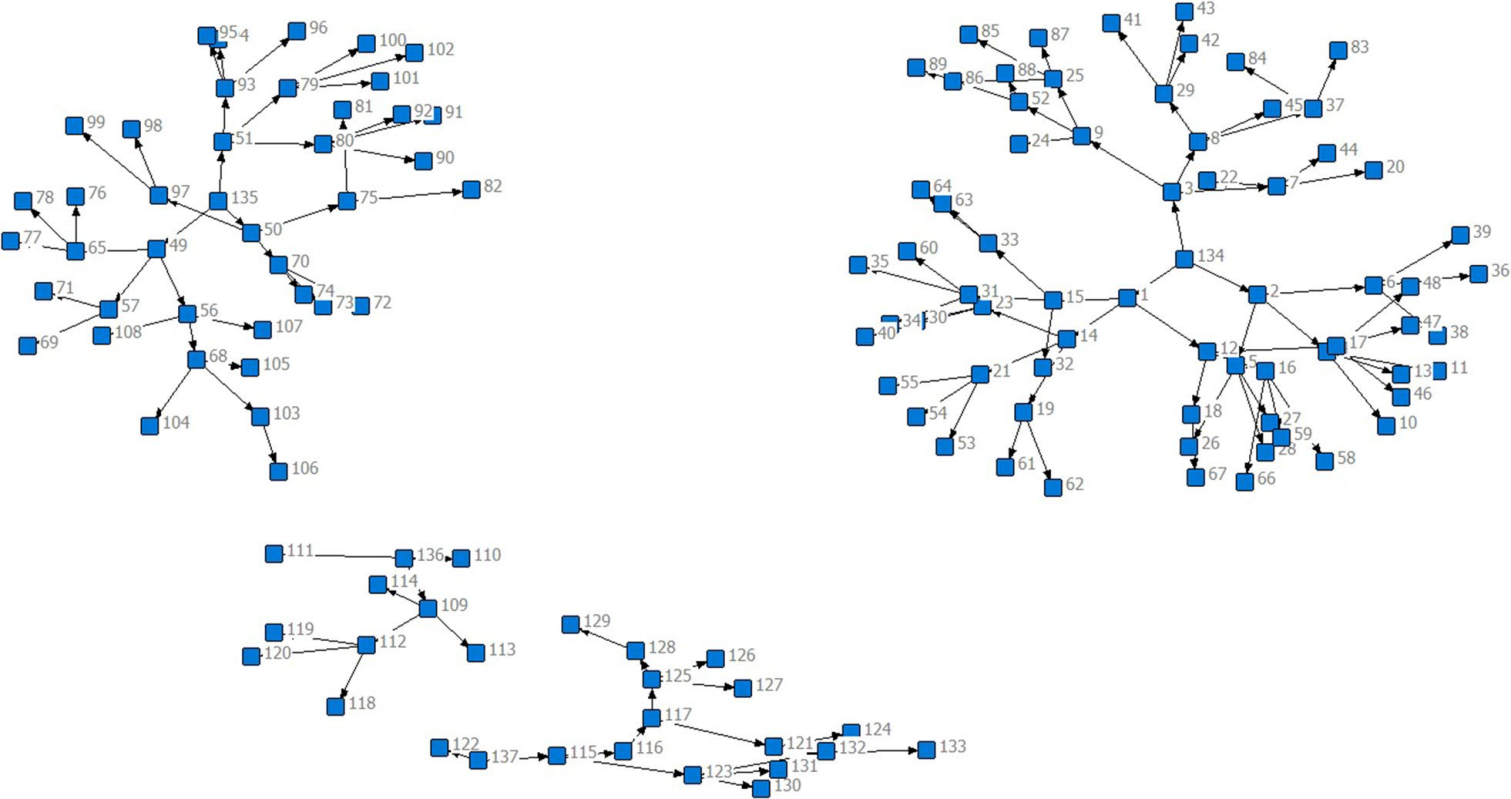
Peer-driven recruitment network from initial seed clients as recruited by the harm reduction organisation “Aceso”, a VCT NGO based primarily in Georgia, with branches in Akhaltsikhe and Borjomi

The PDI sample was mapped for some of the seeds and their recruited peers as part of the recruitment process. The mapped results for two example networks are shown for two of the harm reduction sites involved in RDS in Figs. 1, 2. Mapping was conducted to gain an overview of the linkages and community networks of harm reduction organisations during the recruitment process. The two below examples were randomly selected to demonstrate the mapping process and indicate the level of network interconnection.

The same tool was used to assess knowledge, practice, behaviours, belief and risk in both NSP and PDI groups, which had been used in previous rounds of PDI recruitment. There were 6 survey sections used: demographic data (17 questions), drug-use practice (28 questions), Risk Assessment Battery (29 questions), service assessment (3 questions), HIV knowledge (5 questions) and hepatitis C knowledge (19 questions). The HIV knowledge questions were selected from the United Nations General Assembly Special Session on HIV/AIDS (UNGASS) indicators and adapted to local cultural conditions, based on willingness to respond during face-to-face interviewing, seen through harm reduction activities in Georgia over the last decade. The same 5 questions were asked in our previous publication. Risk Assessment Battery (RAB) was used in the previous report to define the risk undertaken in terms of risky drug and sex behaviour, indicating an average score out of a possibly 40 points, whereby individual risk is tallied, with an average risk score able to be derived for the risk categories. These scores can be averaged across demographic groups. Further information on RAB analysis follows below. Interviewing took 20—30 min. JotForm online data entry form was used to collect survey data from all participant organisations. JotForm’s online form, by automating complex tasks, ensured the minimisation of data entry errors.

The collected data were downloaded and merged into one dataset in Microsoft Excel and later underwent statistical analysis with IBM Statistical Package for Social Sciences (SPSS), versions 21 and 26. Participants whose data had not been entered by the time of analysis were not included. “Refused to answer” answers were included in the analysis (as most presented data refers to a positive response). A Chi-square test was used for categorical data and independent t-test for continuous data. RAB index values were compared by one-way ANOVA, comparing for each characteristic in NSP and PDI samples separately and then as an aggregate. The RAB index results underwent a multiple linear regression to assess the contribution of surveyed factors and their contribution to risk score outcomes. The regression used stepwise entry. Regression was applied to the overall score, and not the individual drug and sex scores as these are products of the overall score. Statistical significance was considered for *p* values < 0.05.

To test the study hypothesis that the peer-recruited population were significantly different and had different risk tendencies to the client population, it was assumed that PWIDs with more than 6 months NSP program experience would have familiarity with harm reduction programmes, and their knowledge of risky drug-use behaviours, transmission risks and sexual risks, and risk characteristics such as injecting behaviours, syringe sharing, risky sexual behaviours and overdose frequency. The results of the known population could be compared to RDS-recruited PWIDs, who had no previous access to harm reduction programmes in the year prior and the education that comes with involvement, and the results could show whether differences did indeed exist. To increase the chance of identifying differences between those currently using harm reduction services, and those not, NSP participants have to have been using services for greater than 6 months, and RDS participants had to have not used any such service in at least 1 year. For NSP users, 6 months was used as a cut-off as this was agreed as an adequate time to achieve basic harm reduction education, to participate in 3−5 of both counsellor and peer education sessions, and to use sterile paraphernalia. The results of the two groups were aggregated and averaged to allow comparison between the two groups of very different sizes.

## Results

A total of 2807 participants were recruited. Of these, 987 were participants already known to NSP services. In the PDI sample, 1820 participants were recruited, from an initial 54 seed recruiters. A total of 5998 coupons had been issued, with 1990 returned (33%). Data were incomplete for 170 participants linked to a coupon number, giving a total of 1820 valid entries. Tbilisi was the most represented city in the survey (It also has the largest population as the capital). The mean age in the NSP group was 41.5, compared to 35.3 in the PDI group, and age range was 18−73. Differences in average age are likely due to designed incentive to recruit young people through RDS. Age of first injection was younger in the PDI group (19.5 vs 20.0) with a range of 10 to 49 (however, it is possible that this could be linked to the purposeful recruitment and incentivisation of young people to this group). Young people were much more represented in the PDI group compared to the NSP group (25.1% vs 3.2%), due to recruitment design and incentivisation. Females were also more represented in the PDI group (6.9% vs 2.0%), again due to design and incentivisation. Most participants reported being heterosexual, and differences were not statistically significant. Statistically significant differences were seen in subgroups in terms of education, housing conditions and medically assisted treatment.

Full descriptive statistics are available in Tables 1 and 2. Not all questions and results are included in Tables 1 and 2; a number of questions asked about services availability and were not included in this review. Questions and answers were also not included if they were considered negligible value, or had very few relevant answers.

**Table 1:** Participants’ socio-demographic characteristics (NSP *n* = 987; PDI *n* = 1820)

Characteristic	NSP: *n* (%^a^) *n* = 987	PDI: *n* (%^a^) *n* = 1820	*P* value
*Demographics*			
City			
Akhaltsikhe (*n* = 140)	0 (0.0)	140 (5.0)	–
Batumi (*n* = 195)	74 (2.6)	121 (4.3)	
Borjomi (*n* = 171)	6 (0.2)	165 (5.9)	
Gori (*n* = 95)	95 (3.4)	0 (0.0)	
Kutaisi (*n* = 278)	76 (2.7)	202 (7.2)	
Ozurgeti (*n* = 221)	63 (2.2)	158 (5.6)	
Poti (*n* = 203)	55 (2.0)	148 (5.3)	
Rustavi (*n* = 213)	100 (3.6)	113 (4.0)	
Samtredia (*n* = 184)	71 (2.5)	113 (4.0)	
Tbilisi (*n* = 754)	295 (10.5)	459 (16.4)	
Telavi (*n* = 135)	70 (2.5)	65 (2.3)	
Zugdidi (*n* = 217)	81 (2.9)	136 (7.5)	
Age^c^(note: recruitment of young people was a specific goal of the RDS) (range 18–73)			
18–25	32 (3.2)	456 (25.1)	**0.00**
> 26	955 (96.8)	1364 (74.9)	
Mean^a^	41.5	35.3	**0.00**
Sex^c^(note: recruitment of females was a specific goal of the RDS)			
Female (*n* = 145)	20 (2.0)	125 (6.9)	**0.00**
Male (*n* = 2662)	967 (98.0)	1695 (93.1)	
Sexual orientation^a^			
Heterosexual	986 (99.9)	1815 (99.7)	0.34
Bisexual	1 (0.1)	1 (0.1)	0.66
Homosexual	0 (0.0)	4 (0.2)	0.14
Education^a^			
Secondary (incomplete)	74 (7.5)	163 (9.0)	0.19
Secondary (complete)	467 (47.3)	839 (46.1)	0.49
Tertiary (incomplete)	132 (13.4)	303 (16.6)	**0.02**
Tertiary (complete)	161 (16.3)	186 (10.2)	**0.00**
Professional	149 (15.1)	327 (18.0)	0.06
Employment^a^			
Unemployed	563 (57.0)	1105 (60.7)	0.06
Self-employed	236 (23.9)	396 (21.8)	0.19
Temporary work	119 (12.1)	202 (11.1)	0.45
Full-time work	63 (6.4)	93 (5.1)	0.16
Pensioner	1 (0.1)	5 (0.3)	0.34
Income source^b,c^			
Employment	366 (37.1)	610 (33.5)	0.56
Renting or selling	135 (13.7)	269 (14.8)	0.43
Friends, relatives, partners	295 (29.9)	539 (29.6)	0.88
Welfare, pension	48 (4.9)	74 (4.1)	0.32
Illegal activities	23 (2.3)	36 (2.0)	0.53
Gambling	55 (5.6)	142 (7.8)	**0.03**
Living conditions^c^			
Own apartment	384 (38.9)	541 (29.7)	**0.00**
Apartment sharing	486 (49.2)	1043 (57.3)	**0.00**
Renting	98 (9.9)	193 (10.6)	0.58
Shelter	10 (1.0)	12 (0.7)	0.31
Homeless	6 (0.6)	12 (0.7)	0.87
Previous or current medication-assisted treatment^c^			
Yes	304 (30.8)	215 (11.8)	**0.00**
Currently being treated	116 (11.8)	117 (6.4)	**0.00**

Statistical significance was classified as a *P* value < 0.05 and are in bold

Chi-square test was applied for categorical data, and independent *t *test for continuous data

^a^Unless otherwise specified

^b^In the last 30 days

^c^As a percentage proportion by recruitment group (NSP vs PDI)

**Table 2:** Risk-related behaviours, HIV-related knowledge and history of HIV and HCV testing (NSP *n* = 987; PDI *n* = 1820)

	NSP	PDI	*p* value
*Drug-use behaviours, including drugs injected*			
Age of first injection^a^ (mean)	20.0	19.5	**0.00**
Injections per day^a,b^ (mean)	1.4	2.1	0.23
Injection days^a,b^ (mean)	17.7	13.8	**0.00**
Injection group size^a,b^ (mean)	3.9	3.5	**0.02**
*Drugs used in the past 30 days*^d^			
Heroin/sirets	58.3	54.9	**0.00**
Fentanyl	1.2	2.0	**0.00**
Street methadone	9.9	13.2	**0.00**
Methadone diverted from opioid substitute therapy	5.5	7.1	**0.00**
Street buprenorphine	30.0	36.5	**0.00**
Buprenorphine diverted from opioid substitute therapy	34.2	20.0	**0.00**
Vint (homemade amphetamines)	15.5	12.7	**0.00**
Ephedrine	32.3	23.5	**0.00**
Amphetamines	5.5	2.3	**0.00**
Antihistamines in mixture	7.7	2.7	**0.00**
Overdose in the last 30 days	3.6	6.4	**0.00**
*Drug- and sex-related risky behaviours (behaviours over the past 6 months)*			
Shared equipment	33.4	52.0	**0.00**
Shared syringe	15.3	33.6	**0.00**
Used a syringe after someone	4.6	26.3	**0.00**
Someone used syringe after	6.4	25.4	**0.00**
Shared syringe with someone HIV positive	0.5	2.9	**0.00**
Always used new syringe	77.1	68.1	-
Shared instruments	35.2	53.2	**0.00**
Exchange sex for drugs	1.9	1.6	0.65
Exchange drugs for sex	3.1	1.8	0.05
Exchanged sex for money	0.7	0.9	0.82
Exchanged money for sex	6.4	6.9	0.63
Sex with someone HIV positive	0.0	0.6	0.05
Past number of blood tests for HIV?^a^	3.1	0.6	**0.00**
Never tested for HIV	0.6	67.0	**0.00**
*HIV knowledge (correct answers)*			
HIV risk is reduced if you have only one partner	98.9	98.8	0.61
HIV risk is reduced if a condom is always used	99.5	98.7	0.13
A person with HIV can look healthy	91.2	86.8	**0.00**
HIV can be caught by sharing food or water with an infected person	98.2	98.1	0.94
HIV can be transmitted by mosquito bite	93.1	91.0	**0.00**
*HIV and HCV testing*			
Ever tested for HCV	99.5	34.2	**0.00**
HIV positive on point-of-care testing at interview	0.8	0.4	0.14

Statistical significance was classified as a *P* value < 0.05 and are in bold

Chi-square test was applied for categorical data, and independent *t *test for continuous data. Only percentages given due to overlap between groups

NB: drug types were not included if both groups had less than 5% use

^a^Unless otherwise specified

^b^In the last 30 days

^c^As a percentage proportion by recruitment group (NSP vs PDI)

^d^Use of multiple substances gives cumulative prevalence of greater than 100%

Stark differences were seen in drug-related behaviours between those who had had harm reduction exposure (NSP) and those that had not (PDI). There were differences in the types of drugs used, with more PDI respondents injecting methadone and buprenorphine. The NSP group reported use of most other drugs, significantly heroin, suboxone, vint (home-made amphetamines), ephedrine, amphetamines and antihistamines in mixture, more frequently. The PDI group reported injecting less days in the past month, and in smaller groups, both of which were significantly different from the NSP group. However, they reported double the rate of episodes of intoxication that they termed as drug-induced overdose (6.4% vs 3.6%). This group was, on average, younger, and could represent newer drug users with less experience on dosage. Risk behaviour showed large, significant differences between the PDI and NSP groups, with PDI partaking in a much higher rate of risky activities. The PDI group shared syringes, equipment and instruments at a much higher rate than the NSP group sharing syringes at nearly double the rate of the NSP group (33.6% vs 15.3%) and had shared a syringe with someone with HIV nearly 6 times more frequently (2.9% vs 0.5%). Some of these differences could be influenced by the younger age of the PDI group; however, risk remains in further analysis (see below). The largest difference, however, was seen in HIV testing; 67% of the PDI group reported never being tested for HIV, compared to just 0.6% of the NSP group. However, point-of-care testing for HIV during data collection interview showed no significant difference in HIV diagnosis (PDI 0.4% vs NSP 0.8%, *p *= 0.14).

There was less divergence in HIV knowledge. Out of 5 standardised questions, only two showed a statistically significant difference in the rates of being answered correctly (“can a person with HIV look healthy?” and “can HIV be transmitted by mosquito bite?”). Though the differences were significant for two questions, the difference in the rate of correct answers was still less than 5% between the two groups.

The Risk Assessment Battery results showed differences in risk behaviours between the NSP and PDI groups. The results of selected characteristics, the drug, sex and overall risk, across the NSP and PDI groups, are shown in Table 3. The scores were derived by calculating the total risk score for each individual, based on a risk behaviour and the frequency with which this behaviour was partaken in (“never”, “few times”, “several times”, “one or two times a week”), and then, mean risk score for each participant was derived by dividing an individual’s score by the number of questions. Different groups’ scores were average as below and compared between other variables. The scores reported are the group average score. Table 3 compares results between different responses within the same group (NSP or PDI).

**Table 3:** Drug, sex and overall risk index for selected demographics across NSP and PDI groups (95% confidence intervals and *p *values)

Characteristic		Sample group											
Groups		NSP						PDI					
		Overall drug index	*p *value	Overall sex index	*p *value	Overall risk index	*p *value	Overall drug index	*p *value	Overall sex index	*p *value	Overall risk index	*p *value
Age	< 25	.50 [.46–.53]	.67	.70 [.67–.74]	**.01**	.59 [.56–.62]	.14	.49 [.48–.50	.96	.71 [.70–.71]	**.00**	.59 [.58–.59]	**.00**
	> 26	.49 [.48–.49]		.67 [.66–.67]		.57 [.56–.57]		.49 [.48–.49]		.66 [.66–.67]		.57 [.56–.57]	
Sex	Female	.47 [.42–.51]	.42	.63 [.60–.67]	**.04**	.54 [.51–.57]	.11	.50 [.49–.52]	.10	.66 [.64–.67]	**.04**	.57 [.56–.59]	.88
	Male	.49 [.48–.50]		.67 [.66–.67]		.57 [.56–.57]		.49 [.48–.49]		.67 [.67–.68]		.57 [.57–.57]	
Education	Secondary	.49 [.48–.50	.71	.67 [.66–.68]	.17	.57 [.56–.58]	.35	.49 [.48–.50]	.37	.67 [.66–.68	.15	.57 [.57–.58]	.91
	Higher	.49 [.47–.50]		.66 [.66–.67]		.57 [.56–.57]		.48 [.48–.49]		.68 [.67–.68]		.57 [.57–.58]	
Overdose in the past 30 days	Yes	.58 [.53–.64]	**.00**	.71 [.70–.75]	**.00**	.64 [.60–.68]	**.00**	.51 [.49–.54]	0.20	.70 [.68–.72]	**.01**	.60 [.58–.61]	**.00**
	No	.48 [.48–.49]		.67 [.66–.67]		.57 [.56–.57]		.49 [.48–.49]		.67 [.67–.68]		.57 [.57-.57]	
Syringe use in the last 6 months	Always used a new syringe	.48 [.48–.49]	.05	.67 [.67–.68]	**.03**	.57 [.56–.57]	.60	.46 [.46–.47]	**.00**	.67 [.66–.67]	**.00**	.56 [.55–.56]	**.00**
	Re-used syringes	.50 [.48–.52]		.68 [.67–.68]		.60 [.59–.60]		.54 [.53–.55]		.68 [.68–.69]		.60 [.60–.61]	
Condom use in the last 6 months	Always	.49 [.47–.50]	.86	.63 [.62–.64]	**.00**	.55 [.54–.56]	**.00**	.48 [.47–.49]	.28	.62 [.61–.63]	**.00**	.54 [.54–.55]	**.00**
	Not always	.49 [.48–.49]		.69 [.68–.69]		.58 [.57–.58]		.49 [.48–.50]		.68 [.68-.69]		.58 [.57–.58]	
Level of worry about HIV	Not bothered	.51 [.49–.53]	**.00**	.66 [.65–.67]	.41	.58 [.57–.59]	**.00**	.47 [.46-.49]	**.00**	.65 [.64–.66]	**.00**	.55 [.54–.56]	**.00**
	Somewhat	.49 [.48–.50]		.67 [.66–.68]		.57 [.56–.59]		.50 [.49–.50]		.67 [.66–.67]		.57 [.57–.58]	
	Significantly	.45 [.43–.46]		.67 [.66–.68]		.55 [.54–.55]		.48 [.47–.49]		.70 [.70–.71]		.58 [.57–.59]	
HIV knowledge	Yes	.48 [.48–.49]	**.00**	.67 [.66–.67]	.26	.58 [.56–.60]	.08	.49 [.49–.50]	**.00**	.67 [.67–.68]	.34	.58 [.57–.58]	**.00**
	No	.51 [.47–.54]		.66 [.65–.67]		.56 [.55–.57]		.46 [.45–.47]		.67 [.66–.68]		.55 [.55–.56]	
Tested for HIV previously and know the result	Never tested	.34 [–.53–1.21]	.05	.67 [–.39–1.37]	.53	.49 [.33–.65]	.06	.49 [.48–.50]	.05	.68 [.68–.69]	**.00**	.58 [.57–.58]	**.00**
	Tested negative	.49 [.48–.50]		.67 [.66–.67]		.57 [.56–.57]		.48 [.47–.49]		.65 [.64–.66]		.56 [.55–.56]	
	Tested positive	.41 [.23–.59]		.63 [.57–.69]		.51 [.40–.61]		n/a		n/a		n/a	

Statistical significance was classified as a *P* value < 0.05 and are in bold

Scores were derived by standardised risk-related questions, averaged per respondent. One-way ANOVA was used to derive averages and confidence intervals

Differences in risk behaviours were seen between the NSP and PDI samples and within the different groups of responders to RAB. Younger people (under 25) were found in both samples to have a significantly higher sex risk, but non-significant differences in drug risk or overall risk, and this seems to be despite differential recruitment strategies. Women had a significantly lower sex risk in both samples, but non-significant differences in drug or overall risk, again, despite recruitment strategy. Those who had overdosed in the previous 30 days had a significantly higher risk in nearly all 3 categories across both NSP and PDI groups (with the exception of drug risk in the PDI sample). Those in the PDI group who always used new syringes when injecting had a significantly lower risk in all categories, which was not reflected in the NSP sample. Those who always used new syringes in the NSP group only had a significantly lower risk for sex risk. Those who always used condoms in the past 6 months had a significantly lower risk for sex risk and overall risk in both samples. Education beyond secondary school was not associated with any decreased risk.

In terms of specific HIV-focussed questions, fewer differences were seen. In the NSP sample, those who reported being “not bothered” about their personal risk of having HIV showed significantly higher scores for drug and overall risk, whereas in the PDI sample, the opposite was true across all three categories. Scoring 5 correct answers for questions assessing HIV knowledge was associated with a significantly lower drug risk in the NSP group. However, in the PDI group, scoring 5 correct answers in the HIV knowledge questions was associated significantly with higher drug risk and overall risk. Sex risk was the same for those who scored 5 correct answers, and those who didn’t, in the PDI group. Finally, in the NSP group, there were no significant differences between those who had never been tested for HIV, those who had been tested and were negative, and those who had been tested and were positive. However, in the PDI sample, sex risk and overall risk were higher in those never tested, and this was statistically significant. There was no significant difference in drug risk between those never tested and those tested and negative. There were no PDI respondents who had been tested positive in the sample.

Table 4 shows a selected further RAB analysis of characteristics across the entire sample of NSP and PDI combined, as guided by the results of Table 3. When treated as one sample, sex risk, and therefore overall risk, remained higher in the young (age under 25) group, and this was statistically significant. Drug risk was not significantly different. Females had a significantly lower sex risk, but not drug or overall risk. Having had an overdose in the previous 30 days was associated with a significantly higher risk in drug, sex and overall risk categories. Always using a new syringe when injecting was associated with a significantly lower drug, and therefore overall, risk across the aggregated group. Interestingly, scoring 5 correct answers on HIV knowledge questions was associated with a significantly higher drug and therefore overall risk score across both groups. There were no significant differences for sex risk.

**Table 4:** Drug, sex and overall risk across total group for selected characteristics

Characteristic		Overall					
Groups		Overall drug index	*p *value	Overall sex index	*p *value	Overall risk index	*p *value
Age	< 25	.49 [.48–.50]	.89	.71 [.70–.71]	**.00**	.59 [.58–.59]	**.00**
	> 26	.49 [.48–.49]		.66 [.66–.67]		.57 [.56–.57]	
Sex	Female	.50 [.48–.52]	.23	.65 [.64–.67]	**.01**	.57 [.56–.58]	.72
	Male	.49 [.48–.49]		.67 [.67–.68]		.57 [.57–.57]	
Overdose in the past 30 days	Yes	.53 [.51–.55]	**.00**	.70 [.68–.72]	**.00**	.61 [.59–.62]	**.00**
	No	.49 [.48–.49]		.67 [.67–.67]		.57 [.56–.57]	
Syringe use in the last 6 months	Always used a new syringe	.47 [.47–.48]	**.00**	.67 [.66–.67]	.05	.56 [.56–.56]	**.00**
	Re-used syringes	.53 [.52–.54]		.68 [.67–.68]		.60 [.59–.60]	
HIV knowledge	Yes	.49 [.49–.49]	**.02**	.67 [.66–.67]	.19	.57 [.57–.57]	**.01**
	No	.48 [.46–.49]		.67 [.67–.67		.56 [.55–.57]	

Statistical significance was classified as a *P* value < 0.05 and are in bold

One-way ANOVA was used to derive averages and confidence intervals

A stepwise multiple linear regression analysis was undertaken with the overall risk as dependent variable and the surveyed demographic details as independent variables. Stepwise regression eliminated gender and education as non-significant variables. Age and city were found to be associated with overall risk scores, with age showing a negative association in the regression model (β = -0.173, p = 0.000). This finding of an association between younger age and risk complements the findings of the RAB analysis. The regression model attributed 39% of the variance in risk to differences in age and city across the entire cohort (R^2^ 0.039, p = 0.00). While not all possible confounding factors were surveyed and able to be analysed as part of the regression model, 61% of the variance in risk remains unexplained, while RAB analysis shows significant differences in risk between the NSP and PDI respondents (Table 5).

**Table 5:** Stepwise multiple linear regression analysis of overall risk score against surveyed demographic factors—model summary

*R*	*R*^2^	Adjusted *R*^2^	Standard error of the estimate
*Model summary*			
.198	.039	.039	.082

	Sum of squares	*df*	Mean squares	*F*	Sig
*ANOVA*					
Regression	.771	2	.385	57.115	0.00
Residual	18.867	2797	.007		
Total	19.637	2799			

	Unstandardised coefficients		Standardised coefficients	*t*	Sig
	*B*	Standard error	Beta		
*Coefficients*					
(Constant)	.607	.006		102.393	.000
Age	− .001	.000	− .173	− 9.308	.000
City	.002	.000	.109	5.890	.000

## Discussion

Significant differences were shown between the clients known to harm reduction programmes across Georgia, and those recruited through peers to the programmes, who hadn’t interacted in the previous 12 months. The recruitment strategy of encouraging recruitment of younger people and females was successful, and significantly higher proportions of these two groups were able to be recruited compared to the sample clients already known to services, shown by statistically significant difference in proportions between the two groups. Younger people showed higher sex risk in the NSP, PDI and overall groups. There were mixed differences in drug-use types across the two groups, but large differences were seen in terms of risk, with the PDI samples having significantly higher rates of risky sharing practices, and much, much lower rates of testing for HIV. Knowledge of HIV was not as divergent as expected between the two groups. There were no significant differences seen in actual HIV status from point-of-care testing between the two groups at the time of interview.

Sampling showed efficacy in recruiting previously unknown clients to harm reduction programs. This was observed in a previous PDI trial reported by the authors. Special incentive to recruit young people and females through the incentivisation of the RDS was successful in this sample. Similar to last time, PDI was effective in recruiting young people under the age of 25 to harm reduction programmes, aided by design. However, in this sample, recruiting women was much more successful, as our prior sampling actually recruited fewer women through PDI sampling. Again, this was a specific design feature, and an incentivised aim of the PDI through RDS. This success is more in line with what has been observed in some of the literature in other ex-Soviet nations, such as in Ukraine, where PDI was efficacious in recruiting a higher proportion of young people and females, compared to normal methods [19]. It is worth noting that the current survey showed that less than 0.3% of respondents reported a sexual orientation other than heterosexual. Usually, prevalence of homosexuality in a society is many times higher than this [20], implying people may be under-reporting their sexual orientations, habits, and potentially, risk.

The sample showed that NSP clients were less likely to practice sharing behaviours during injection and more likely to have been tested for HIV in the past. The difference in testing rates between the NSP and PDI groups in the sample is a cause for concern, with only 33% of PDI respondents saying that they had been previously tested, compared to more than 99% in the NSP group. It would be assumed that these differences were due to the education received when attending harm reduction services and the ability for testing, counselling and referral while attending the services. Part of this difference in risk may be due to drop out of client. It is possible that clients who may have dropped out and have subsequently been re-recruited, have intrinsic differences in risk behaviour than those who remain engaged, as other studies have shown that peer recruitment is able to re-engage clients who may have dropped out [16]. Other studies of drug use and risk, and HIV testing [21], emphasise the importance of recruitment of drug users to harm reduction programmes and potentially the importance of retaining PWIDs in harm reduction engagement.

The results highlight the trends in the dynamic, changing drug-use scene in Georgia. The PDI sample showed significantly lower rates of heroin use and higher rates of use of diverted street drugs, such as methadone and buprenorphine. This has been shown to be a wider trend regionally [19]. Information such as this is critical in directing harm reduction efforts. Differences exist between those in NSP, who may have a higher likelihood of using some traditional drugs of injection, and those who lie outside of these programmes, who may be influenced by other drug culture changes. Other studies support this, such as in the increasing use of diverted medications and adulterated opiates in the USA [22]. The increasing use of fentanyl has been identified as a culture shift in other countries [22], and our sample showed that PDI respondents had significantly higher rates of the use of fentanyl. Fentanyl was not reported in our previous report based on the 2015 sample.

The differences seen in the Risk Assessment Battery are crucial for planning ongoing harm reduction services. Comparing the results of the RAB for the current sample and the 2015 sample illustrates the need for ongoing assessment and update of information provision and intervention target; the previous sample showed lower sex risk in younger people in the PDI group, and no significant differences in the NSP group. This sample, however, showed the opposite, significantly higher sex risk in younger people, in both NSP and PDI groups. Further, the 2015 sample showed lower drug risk and higher sex risk in females in both the NSP sample and lower risk for all three categories in the PDI sample. In the current sample, however, women showed a significantly lower rates of sex risk behaviour across the two groups, which continued when aggregated. The importance of tailored, flexible programmes that meet the needs of clients has been identified in other locations [23], as has the ability of drug harm reduction programmes to impact sex risk [23]. These findings will be vital in guiding harm reduction activities in Georgia into the future, as will the purposeful incentivisation to recruit marginalised young people and females.

It was an unexpected finding that knowledge on HIV did not differ dramatically between the NSP and PDI groups. There were no significant differences between NSP and PDI for 3 of the 5 standardised HIV knowledge questions. Previously published research by the GHRN showed significant differences in HIV knowledge between NSP and PDI clients. It is possible that HIV knowledge has been disseminating through peers in their drug-using networks, independently of respondent-driven sampling for recruitment to programmes. It is also possible that those recruited had previously been involved in harm minimisation activities and that knowledge from previous education had been retained. Details of whether any of the PDI participants were ever previously involved with harm minimisation services were not sought. There was also believed to be an effect on knowledge in the PDI group from interaction with opioid substitution therapy services, and from online platforms, and this may have contributed to the level of knowledge in the respondent-driven sample.

The use of peers in recruiting and guiding harm reduction programmes is widely documented and supported in recent literature [13–16]. Peer leadership has been identified as an asset in building, guiding and implementing new concepts in the realm of public health, such as Whole Systems Approaches, where the interacting, complicated factors that influence individuals are mapped, and interventions planned around managing multiple contributory factors on multiple levels of personal influence simultaneously. A pilot study in Australia of a Whole Systems Approach for HIV and Hepatitis C testing involved peers as an integral part of the harm reduction programme, and supported the use of peers in such programmes into the future [24]. As public health evolves into the future to meet new and evolving challenges, it will be essential that a peer focus is included in considering how to meet these challenges and how best to recruit and serve the client populations, including in drug-related harm reduction.

## Conclusion

The Republic of Georgia continues to be at risk of expanding blood-borne disease epidemics, including related to drug use. Harm reduction activities are vital in reducing morbidity and mortality. Though programmes are continuously growing, social limitations remain, and populations still remain hidden to harm reduction services. Peer-driven interventions, including the use of respondent-driven sampling, are seen as a way to build trust in drug-using populations and involve people previously unknown in harm reduction services to improve public health. The involvement of peers in planning, recruiting and delivering public health services, including around drug harm reduction, is promoted, including in new types of public health interventions. The results show that peer-driven interventions, such as this one using respondent-driven sampling, can be successful in recruiting previously unknown, marginalised populations (such as younger people and females) to harm reduction programmes, and these unknown populations have specific risk behaviours and differences that are important to inform harm reduction programmes into the future. The design of this PDI was able to recruit people with lower testing rates for blood-borne viruses.

Ongoing vulnerabilities and risks exist between different subgroups of the drug-using community. The above findings highlight significant differences between populations known to, and hidden from, harm reduction activities. These have evolved over time and are not the same as previous findings. This underscores the need for ongoing monitoring and evaluation of programmes and the populations which they serve. Realising the dynamic change seen in client populations is essential in guiding and adapting services into the future to best reduce harm and tailor programmes to the client population. This report quantified that different levels continue to exist between people known to harm reduction services and those who remain hidden to them. It speaks to the power of peer-driven interventions in reaching clients not only previously unknown, but also those at different levels of risk, to the known population. This report also underscores the importance of repeated peer-driven interventions and respondent-driven sampling as this intervention was able to recruit high-risk, vulnerable populations that were different to the previous peer-driven interventions, through design and incentivisation, who had significant risk differences. These risks had changed from previous research.

Future directions should further quantify how subsequent PDI programmes can recruit different high-risk populations from previous interventions. Running subsequent programmes seems to be beneficial to the target population; in this sample, people of different risk to previous recruitments were introduced to the harm reduction programmes. There should also be investigation into how these high-risk groups are retained in programmes and the factors that contribute to whether they remain engaged with harm reduction programmes, or if they drop out. It is this evidence-based guidance of dynamic change that is needed in public health programmes. Involvement of peers, building trust, provision of harm reduction materials and education remain important ways to reduce harm in this vulnerable population and improve public health.

### Limitations

There were several limitations that mean the data above should be treated with some caution. The most striking feature was the likely under-reporting of bisexuality or homosexuality. This is most obvious in identification of sexual orientation, as rates of people identifying as non-heterosexuality are known to be higher than what was reported in our sample. Global surveys usually report a prevalence of heterosexuality at around 90% [20], whereas our sample report 99.7% heterosexuality. It does, however, highlight the potential for under-reporting throughout all self-reported data and the potential influence of stigma around sex risk.

Other events reduce the power of our study. The sampled population is not representative of the wider society, nor were people included sequentially. The sample was in people who presented to harm reduction services and consented to participate. A significant number of people did not attend compared to how many coupons were given out, did not have valid answers recorded, or could not be analysed within the time frame of the sampling. It should be noted also that the base population of drug-using individuals is not known in Georgia, so therefore, analytical calculations based on population size, such as test for equilibrium, could not be performed. These all place limitations on the generalisability of the data, and no inferences about population levels of drug use can be made from our data, as it refers to a cohort of drug users.

## Data Availability

The datasets generated and/or analysed during the current study are not publicly available due to the sensitive nature of the data and affiliations within harm reduction organisations of the participants, but are available from the corresponding author on reasonable request.

## Abbreviations

GHRN: Georgian Harm Reduction Network
HCV: Hepatitis C virus
HIV: Human immunodeficiency virus
IDU: Injecting drug user
MSM: Men who have sex with men
NGO: Non-governmental organisation
NSP: Needle syringe program
PDI: Peer-driven intervention
PWID: People who inject drugs
RAB: Risk assessment battery
RDS: Respondent-driven sampling
VCT: Voluntary counselling and testing
WHO: World Health Organization
